# Mutant prevention concentration of orbifloxacin: comparison between *Escherichia coli*, *Pseudomonas aeruginosa*, and *Staphylococcus pseudintermedius* of canine origin

**DOI:** 10.1186/1751-0147-55-37

**Published:** 2013-05-01

**Authors:** Takae Shimizu, Kazuki Harada, Yasushi Kataoka

**Affiliations:** 1 Laboratory of Veterinary Microbiology, Nippon Veterinary and Life Science University, 1-7-1, Kyonan-cho, Musashino, Tokyo, 180-8602, Japan; 2 Department of Veterinary Internal Medicine, Tottori University, Minami 4-101, Koyama-Cho, Tottori-Shi, Tottori, 680-8553, Japan

**Keywords:** Mutant prevention concentration, Fluoroquinolone, *Escherichia coli*, *Pseudomonas aeruginosa*, *Staphylococcus pseudintermedius*, Canines

## Abstract

**Background:**

The mutant prevention concentration (MPC) is an important parameter to evaluate the likelihood of growth of fluoroquinolone-resistant mutants for antimicrobial-pathogen combinations. The MPCs of fluoroquinolones for different canine pathogens have not been compared. In this study, we compared for the first time orbifloxacin MPCs between susceptible strains of *Escherichia coli*, *Pseudomonas aeruginosa*, and *Staphylococcus pseudintermedius* of canine origin.

**Methods:**

More than 10^10^ CFU/ml of 10 strains of each bacterial species were inoculated onto Muller-Hinton agar supplemented with different concentrations of orbifloxacin from 1× to 64× minimum inhibitory concentration (MIC) and the MPCs were recorded. MICs of original strains and of mutants arising after exposure to sub-MPC concentrations (one per original strain) were determined in the presence or absence of efflux pump inhibitors (EPIs). The effects of quinolone resistance-determining region (QRDR) mutations were also examined.

**Results:**

MPCs were significantly higher for *P. aeruginosa* (16–128 μg/ml) than for *E. coli* (0.5–32 μg/ml). MPCs for *S. pseudintermedius* varied between the low-susceptible (16–128 μg/ml) and the high-susceptible strains (4–16 μg/ml) and were the most broadly distributed among the three species. Regarding resistance mechanisms, only one QRDR mutation in *gyrA* was found in all of the 10 mutants of *E. coli* and in 4 of the 10 mutants of *P. aeruginosa*, whereas mutations in both *grlA* and *gyrA* were found in 3 mutants and one mutation in *grlA* was found in 2 mutants among the 10 mutants of *S. pseudintermedius*. In the presence of an EPI, the MICs of *P. aeruginosa* mutants decreased markedly, those of *E. coli* mutants decreased moderately, and those of *S. pseudintermedius* mutants were unaffected.

**Conclusions:**

MPCs of orbifloxacin vary between bacterial species of canine pathogens, possibly due to the diversity of the main fluoroquinolone resistance mechanism among these species. Therefore, the type of bacterial species should be taken into consideration when using fluoroquinolone drugs such as orbifloxacin in canines.

## Background

The incidence of canine pathogens showing decreased susceptibility or increased resistance to fluoroquinolones was reported in Japan [1-3] as well as worldwide. Determination of the mutant prevention concentration (MPC), the antimicrobial concentration that prevents selection of resistant mutants, is important for reduction of the incidence of fluoroquinolone resistance [4]. It is hypothesised that drug exposure below the MPC may promote selection of resistant strains and the concentration range between the minimum inhibitory concentration (MIC) and MPC, which is referred to as the mutant selection window (MSW), may enrich and amplify resistant mutants. Thus, MPC and MSW are important parameters for evaluation of the potential for emergence of fluoroquinolone-resistant mutants for antimicrobial-pathogen combinations [4]. MPCs of fluoroquinolone in canines have been sporadically reported for single pathogens [5-7]. However, a consistent comparison of fluoroquinolone MPCs among different pathogens has not been performed.

Resistance to fluoroquinolone is mediated primarily through mutations in the quinolone resistance-determining region (QRDR) of DNA gyrase and topoisomerase IV [8]. Decreased drug uptake due to overexpression of drug efflux pumps also contributes to development of fluoroquinolone resistance [8].

Orbifloxacin is a fluoroquinolone that was developed for use in veterinary medicine and has achieved a relatively large sales volume (approximately 137 kg in 2011) among fluoroquinolones for companion animals in Japan [9]. This antimicrobial agent exhibits bactericidal activity against numerous Gram-negative and Gram-positive bacteria (e.g. *Escherichia coli*, *Pseudomonas aeruginosa*, and *Staphylococcus pseudintermedius*) and is thus indicated for treatment of canine bacterial infections, including urinary, skin, and ear infections. Gebru et al. [6,7] found that orbifloxacin MPCs were relatively high compared to those of other veterinary fluoroquinolones, which may be helpful in establishing a comprehensive understanding of the variation of MPCs among different pathogens.

The purpose of the present study is to investigate differences in the likelihood of emergence of fluoroquinolone-resistant mutants among major bacterial pathogens based on MPC determination and to characterize the resistance mechanism of mutants. We compared the MPCs of orbifloxacin among fluoroquinolone-susceptible *E. coli*, *P. aeruginosa*, and *S. pseudintermedius* strains. Mutants arising after exposure to sub-MPC concentrations were screened for QRDR mutations and the effects of efflux pump inhibitors (EPIs) on the MICs of orbifloxacin were determined.

## Methods

### Bacterial isolates

Ten fluoroquinolone-susceptible strains each of the following three bacterial species were used in this study: *E. coli* (strains E1–E10), *P. aeruginosa* (strains P1–P10), and *S. pseudintermedius* (strains S1–S10). *E. coli* and *P. aeruginosa* strains were selected from our collection of urine and ear/skin samples, respectively, obtained from domestic dogs [1,2]. *S. pseudintermedius* strains were isolated from swabs obtained from dogs with canine pyoderma at the Veterinary Medical Teaching Hospital, Nippon Veterinary and Life Sciences University, and at three veterinary hospitals located in Tokyo, Japan. Swabs were streaked onto mannitol salt agar (Eiken Chemical, Japan) and typical colonies were collected. Bacterial identification was carried out by Gram staining, catalase and coagulase tests, and multiplex-polymerase chain reaction (PCR) [10]. All confirmed *S. pseudintermedius* isolates were stored at −80°C in 10% skimmed milk.

### Determination of MPCs and mutant recovery

MPCs were determined using a previously described protocol [11] with slight modifications. A concentrated cellular suspension of each bacterial strain (200 μl) containing >10^10^ colony-forming units (CFU)/ml was plated onto each of three Mueller-Hinton agar (Becton Dickinson, France) plates, which were supplemented with orbifloxacin at a concentration equal to the MIC and six doubling dilutions higher than the MIC (i.e. 2×, 4×, 8×, 16×, 32×, and 64× MIC). Plates were incubated at 37°C for 5 days because preliminary tests showed no significant differences in MPCs between incubations for 2 and 5 days, similarly with the previous report [11]. The lowest drug concentration that prevented the emergence of mutants after the 5-day incubation period was recorded as the MPC. Each experiment was performed twice.

A mutant of each original strain (EM1–EM10, PM1–PM10, and SM1–SM10) was randomly selected from plates with a concentration of orbifloxacin that was one dilution (i.e. twofold) lower than the MPC (sub-MPC). Each mutant was cultured on antimicrobial-free agar plates for three serial passages and then stored at −80°C until further analysis.

### Susceptibility testing for orbifloxacin

MICs of orbifloxacin against the original strains and mutants were determined using the agar dilution method, according to the guidelines of the Clinical and Laboratory Standards Institute (CLSI) [12]. MICs of orbifloxacin were also determined in the presence of EPIs: 80 μg/ml of Phe-Arg-β-naphthylamide (PAβN, Sigma-Aldrich, MO, USA) for *E. coli* and *P. aeruginosa*, and 20 μg/ml reserpine (Sigma-Aldrich) for *S. pseudintermedius*. All inoculated agar plates were incubated at 35°C for 16–20 h. *E. coli* ATCC 25922, *P. aeruginosa* ATCC27853, *S. aureus* ATCC29213, and *Enterococcus faecalis* ATCC29212 were used as quality control strains.

### PCR amplification and DNA sequencing of QRDRs

The QRDRs of the *gyrA* and *parC* genes for *E. coli* and *P. aeruginosa* or of the *grlA* and *gyrA* genes for *S. pseudintermedius* in the original strains and in representative mutants of each original strain were amplified by PCR using previously described primers [13-15]. The amplicons were bidirectionally sequenced using the PCR primers.

### Statistical analysis

One-way analysis of variance (ANOVA) was used to compare MPCs and MPC/MIC, serum maximum concentration (C_max_)/MPC, and area under the concentration time-curve (AUC)/MPC ratios among the three bacterial species, based on the results for ten original isolates per species. A Tukey test was used to evaluate differences among the geometric means of these parameters. A Welch test was used for pairwise comparison of MICs. The threshold for significance was set at a value of *P* < 0.05 in all analyses.

## Results

### MICs of original strains and mutants in the presence or absence of EPIs

The results of the study are summarised in Table 1. The MICs of orbifloxacin against the original strains and mutants were 0.063–2 μg/ml and 1–8 μg/ml, respectively, for *E. coli*, and 1–4 μg/ml and 16–128 μg/ml, respectively, for *P. aeruginosa*. Thus, the orbifloxacin MICs against the original strains of *P. aeruginosa* were significantly increased by drug exposure compared with those of the *E. coli* original strains (4- to 32-fold vs. 2- to 16-fold, *P* < 0.05).

**Table 1:** **MICs and MPCs of orbifloxacin and QRDR mutations in the *gyrA*, *parC*, and *grlA *genes of the original stains and mutants used in the study**

**Parent strains and mutants**^**a**^	**MIC (μg/ml)**	**MIC (+EPI)**^**b **^**(μg/ml)**	**QRDR mutation**^**c**^		**MPC (μg/ml)**	**MPC /MIC**	**C**_**max **_**/MPC**^**d**^	**AUC /MPC**^**d**^
			***gyrA***	***parC *( *grlA *)**				
*E. coli*								
E1	0.063	<0.015	wt	wt	1	16	6.9	42.9
E2	0.063	<0.015	wt	wt	0.5	8	13.8	85.8
E3	0.063	<0.015	wt	wt	1	16	6.9	42.9
E4	0.125	<0.015	wt	wt	2	16	3.45	21.45
E5	0.125	<0.015	wt	wt	2	16	3.45	21.45
E6	0.25	<0.015	wt	wt	1	4	6.9	42.9
E7	0.5	0.063	S83L	wt	16	32	0.43	2.68
E8	1	0.063	D87N	wt	8	8	0.86	5.36
E9	2	0.125	D87N	wt	8	4	0.86	5.36
E10	2	0.125	S83L	wt	32	16	0.22	1.34
EM1 (0.5)	1	0.125	S83L	wt	-	-		
EM2 (0.25)	1	0.125	S83L	wt	-	-		
EM3 (0.5)	1	0.125	S83L	wt	-	-		
EM4 (1)	2	0.125	S83L	wt	-	-		
EM5 (1)	2	0.25	S83L	wt	-	-		
EM6 (0.5)	2	0.125	S83L	wt	-	-		
EM7 (8)	4	0.125	S83L	wt	-	-		
EM8 (4)	4	0.25	D87N	wt	-	-		
EM9 (4)	8	0.25	D87N	wt	-	-		
EM10 (16)	4	0.125	S83L	wt	-	-		
*P. aeruginosa*								
P1	1	0.015	wt	wt	32	32	0.22	1.34
P2	1	0.031	wt	wt	16	16	0.43	2.68
P3	1	0.063	wt	wt	32	32	0.22	1.34
P4	2	0.125	wt	wt	64	32	0.11	0.67
P5	2	0.063	wt	wt	32	16	0.22	1.34
P6	2	0.125	wt	wt	32	16	0.22	1.34
P7	2	0.031	wt	wt	64	16	0.11	0.67
P8	2	0.063	wt	wt	128	64	0.05	0.34
P9	4	0.25	wt	wt	64	16	0.11	0.67
P10	4	0.125	wt	wt	64	16	0.11	0.67
PM1 (16)	32	0.063	wt	wt	-	-		
PM2 (8)	16	0.25	wt	wt	-	-		
PM3 (16)	16	0.5	wt	wt	-	-		
PM4 (32)	32	1	T83I	wt	-	-		
PM5 (16)	32	0.25	wt	wt	-	-		
PM6 (16)	64	0.125	wt	wt	-	-		
PM7 (32)	64	1	T83I	wt	-	-		
PM8 (64)	64	0.125	T83I	wt	-	-		
PM9 (32)	128	2	T83I	wt	-	-		
PM10 (32)	16	0.25	wt	wt	-	-		
*S. pseudintermedius*								
S1	0.25	0.25	wt	wt	2	8	3.45	21.45
S2	0.25	0.5	wt	wt	2	4	3.45	21.45
S3	0.25	0.5	wt	wt	8	16	0.86	5.36
S4	0.5	0.5	wt	wt	2	8	3.45	21.45
S5	0.5	0.5	wt	wt	4	8	1.73	10.73
S6	0.5	0.5	wt	wt	4	4	1.73	10.73
S7	1	1	wt	S80I	128	128	0.05	0.34
S8	1	1	wt	S80I	128	128	0.05	0.34
S9	1	1	wt	S80I	64	64	0.11	0.67
S10	1	1	wt	S80I	16	16	0.43	2.68
SM1 (1)	1	1	wt	wt	-	-		
SM2 (1)	0.5	0.5	wt	wt	-	-		
SM3 (4)	1	1	wt	wt	-	-		
SM4 (1)	2	1	wt	wt	-	-		
SM5 (2)	0.5	0.5	wt	wt	-	-		
SM6 (2)	1	2	wt	S80I	-	-		
SM7 (64)	64	128	S84W	S80I	-	-		
SM8 (64)	64	128	S84L	S80I	-	-		
SM9 (32)	8	8	wt	S80I	-	-		
SM10 (8)	32	32	S84L	S80I	-	-		

^a^ Number in parenthesis indicates the orbifloxacin concentration (μg/ml) supplemented in the agar from which mutants were derived.

^b^ Minimum inhibitory concentrations (MICs) in the presence of efflux pump inhibitors (EPIs).

^c^*parC* of *E. coli* and *P. aeruginosa*, and *grlA* of *S. pseudintermedius*; wt, wild type; S83L, Ser-83 → Leu; D87N, Asp-87 → Asn; T83I, Thr-83 → Ile; S80I, Ser-80 → Ile; S84W, Ser84 → Trp; S84L, Ser84 → Leu.

^d^ Data for maximum concentration (C_max_: 6.9 mg/l) and area under the concentration time-curve (AUC: 42.9 mg.h/l) of orbifloxacin (dose of 7.5 mg/kg) are from reference [16].

For *S. pseudintermedius*, the MICs of orbifloxacin against the original strains were 0.25–1 μg/ml. After drug exposure, the MICs of the high-susceptible strains (S1–S6) increased 1- to 4-fold, whereas those of the low-susceptible strains (S7–S10) increased 8- to 64-fold. The MICs of orbifloxacin against the mutants of this species were widely distributed (0.5–64 μg/ml).

Addition of PAβN, an EPI, resulted in a decrease in the MICs of orbifloxacin against the mutants and original strains of *E. coli* and *P. aeruginosa* (*P* < 0.05) by 8- to 32-fold and 32- to 512-fold, respectively. In contrast, the MICs of orbifloxacin against the original strains and mutants of *S. pseudintermedius* were unaffected by addition of reserpine (*P* > 0.05). By comparison of the MICs of mutants for all three bacterial species, the decrease in the MIC of orbifloxacin against *P. aeruginosa* was more pronounced, compared with those for *E. coli* and *S. pseudintermedius* (*P* < 0.05).

### QRDR mutations in original strains and mutants

Sequence analysis of QRDRs revealed that four low-susceptible original strains (E7–E10; MIC: 0.5–2 μg/ml) and all the mutants of *E. coli* harboured one point mutation (Ser-83 → Leu or Asp-87 → Asn) in *gyrA*. In *P. aeruginosa*, four strains (PM4, PM7–PM9) harboured one point mutation (Thr-83 → Ile). No mutations were found in the *parC* gene of *E. coli* or *P. aeruginosa*.

In *S. pseudintermedius*, four low-susceptible original strains (S7–S10; MIC: 1 μg/ml) and four mutants (SM6–SM10) harboured one point mutation (i.e. Ser-80 → Ile) in *grlA*. Of these mutants, three high-level resistant mutants (strains SM7, SM8, and SM10) harboured an additional mutation (i.e. Ser84 → Trp or Leu) in *gyrA*.

Some original strains (i.e. E10, S2 and S5) gave atypical mutants without significant increases in MICs and an additional QRDR mutation emerged after drug exposure, indicating that these original strains have lower incidence of mutations.

### MPCs and MPC/MIC ratios

The MPCs and MPC/MIC ratios for the original strains of *E. coli* were 0.5–32 μg/ml and 4–32, respectively. In this species, low-susceptible strains with one QRDR mutation (strains E7–E10) had relatively higher MPCs (8–32 μg/ml), compared with high-susceptible strains (0.5–2 μg/ml). *P. aeruginosa* exhibited similar MPCs (16–128 μg/ml) and MPC/MIC ratios (16–64) for all original strains. In *S. pseudintermedius*, the MPCs (16–128 μg/ml) and MPC/MIC ratios (16–128) in low-susceptible strains (S7–S10) with one QRDR mutation were higher than those in high-susceptible strains without QRDR mutations (S1–S6; MPC: 2–8 μg/ml and MPC/MIC: 4–16).

According to the published pharmacokinetic data of orbifloxacin, the C_max_ and AUC of orbifloxacin at a dose of 7.5 mg/kg are 6.9 mg/l and 42.9 mg.h/l, respectively, were obtained [16]. Using these data, the C_max_/MPC and AUC/MPC ratios were calculated for *E. coli*, *P. aeruginosa*, and *S. pseudintermedius* as 0.22–13.8 (C_max_/MPC) and 1.34–85.8 (AUC/MPC), 0.05–0.43 and 0.34–2.68, and 0.05–3.45 and 0.34–21.45, respectively. A comparison among the bacterial species showed that MPC was significantly higher, but the AUC/MPC and C_max_/MPC ratios were significantly lower, for *P. aeruginosa* compared to *E. coli* (*P* < 0.05). There were no significant differences in these values between *S. pseudintermedius* and the other two bacterial species. There were also no significant differences in the MPC/MIC ratios among the three bacterial species.

## Discussion

Since introduction of the concept of the MPC, there have been numerous reports of MPCs for fluoroquinolones against Gram-positive and Gram-negative bacteria, but no comparisons of MPCs of fluoroquinolones against different bacterial species under the same experimental conditions. Thus, this is the first comparison of the MPCs of fluoroquinolones against canine pathogens, and the first determination of the MPC of orbifloxacin against *P. aeruginosa*.

Our results showed that the MPC of orbifloxacin against *P. aeruginosa* is higher than that against *E. coli*. Pasquali et al. [11] also found that the MPCs of enrofloxacin and ciprofloxacin are higher against *P. aeruginosa* than against *E. coli*. Collectively, these results indicate that *P. aeruginosa* has a tendency to exhibit higher MPCs for various drugs compared with *E. coli*. In contrast, the orbifloxacin MPCs against *S. pseudintermedius* did not differ significantly from those of *E. coli* and *P. aeruginosa*. This result may be explained by the considerable variation in MPCs among the strains of *S. pseudintermedius*. Awji et al. [7] also found that *S. pseudintermedius* exhibited a wider range of orbifloxacin MPCs, compared with those for other veterinary fluoroquinolones. Therefore, the variable MPCs among *S. pseudintermedius* strains are likely to be due to the type of bacterial species and the susceptibility of the pathogen to orbifloxacin.

To examine the basis for the differences in MPCs of orbifloxacin among the three bacterial species, we determined the MICs and fluoroquinolone-resistance mechanisms in MPC mutants of each species. In this study, high-susceptible strains of *E. coli* lacking a QRDR mutation and low-susceptible strains with one QRDR mutation were used as original strains. The MICs of orbifloxacin against all mutants were categorised as susceptible or intermediate based on the CLSI breakpoint criteria for orbifloxacin (MIC ≥8 μg/ml) [12], except for one strain (strain E9), which exhibited a MIC of 8 μg/ml. Sequence analysis revealed that all *E. coli* mutants harboured only one QRDR mutation in the *gyrA* gene, as also found by Gebru et al. [5]. The two types of *gyrA* mutations found in the current study (S83L and D87N) are known to cause elevated fluoroquinolone MICs in *E. coli*[17,18]. Generally, MICs of fluoroquinolone against *E. coli* increase in correspondence to the number of QRDR mutations [19], which is the primary mechanism for fluoroquinolone resistance [20]. The emergence of only one QRDR mutation in *E. coli* in this study may be mainly responsible for the failure to acquire orbifloxacin resistance. Similarly, several studies have shown that most *E. coli* mutants from MPC plates had one or none of QRDR mutations even when parent strains with one *gyrA* mutation were used [5,6,11]. These findings imply that *E. coli* rarely acquires two or more QRDR mutations in MPC experiments performed under static conditions. In contrast, all strains of *P. aeruginosa* exhibited higher orbifloxacin MICs than the CLSI breakpoint and the MICs were significantly higher than those against *E. coli*. However, a T83I mutation, which elevates fluoroquinolone MICs [1,13], was detected in fewer mutants of *P. aeruginosa*, compared with *E. coli*, and there were no differences in MICs between *P. aeruginosa* mutants with and without QRDR mutation. These findings suggest that QRDR mutations in *P. aeruginosa* play an insignificant role in the increased MICs of orbifloxacin against the mutants and increased MPC of orbifloxacin.

MICs of orbifloxacin for *P. aeruginosa* and *E. coli* mutants were significantly decreased by addition of an EPI (PAβN) but the effect of this EPI was greater on *P. aeruginosa* strains than on *E. coli* strains. Pasquali and Manfreda [11] similarly found that the decreases in the MICs of enrofloxacin and ciprofloxacin in the presence of PAβN were more pronounced for *P. aeruginosa* than for *E. coli*. We previously showed that efflux pumps, rather than QRDR mutations, play an important role in the development of fluoroquinolone resistance in *P. aeruginosa*[1]*.* Differential expression of efflux pumps in *E. coli* and *P. aeruginosa* is likely to be the main factor in the variable increases in orbifloxacin MIC values against mutants of *E. coli* and *P. aeruginosa* and in orbifloxacin MPCs against these two species.

In *S. pseudintermedius*, unlike *E. coli* and *P. aeruginosa*, orbifloxacin MICs against the mutants and MPCs against the original strains differed markedly based on the susceptibility of the original strain. Sequence analysis revealed three types of QRDR mutations at codon 80 of *grlA* and codon 84 of *gyrA*, which are hotspots for mutations that decrease fluoroquinolone susceptibility in *Staphylococcus* spp., including *S. pseudintermedius*[3,8]. High-susceptible original strains lacking the QRDR mutation yielded relatively low orbifloxacin MICs for mutants, which resulted in relatively low MPCs for original strains. In contrast, low-susceptible strains with one QRDR mutation mostly exhibited an additional QRDR mutation after drug exposure and yielded relatively high MICs for mutants, which resulted in relatively high MPCs for original strains. For *S. pseudintermedius*, the relationship between fluoroquinolone susceptibility of the original strain and the MPC value of orbifloxacin has not been investigated previously. Our results imply that fluoroquinolone susceptibility and the status of QRDR mutations in the original strains can greatly affect the MICs of orbifloxacin against mutants and MPC values for original strains. Further studies are needed to explore these findings. Addition of an EPI did not significantly affect the MICs of orbifloxacin against *S. pseudintermedius* mutants, consistent with the results of Awji et al. [7]. These findings suggest that efflux pumps are not responsible for conferring fluoroquinolone resistance in *S. pseudintermedius*.

Conversion of *in vitro* MPCs into clinically useful data requires use of pharmacokinetic and pharmacodynamic parameters of a drug. The C_max_/MPC and AUC/MPC ratios are important predictors for prevention of the emergence of resistant bacteria. In this study, we obtained these parameters based on published C_max_ and AUC values for orbifloxacin in dogs [16] and found that both C_max_/MPC and AUC/MPC for orbifloxacin were lower in *P. aeruginosa* than in *E. coli*, although the values in these two species did not differ significantly from those of *S. pseudintermedius*. Olofsson et al. [21] suggested that an AUC/MPC ratio ≥22 is predictive of prevention of emergence of a fluoroquinolone-resistant mutant. Thus, our data may imply that appropriate orbifloxacin AUC/MPC ratios cannot be achieved, especially in low-susceptible strains of *E. coli* and *S. pseudintermedius*, and in *P. aeruginosa* strains. However, the orbifloxacin concentration may be higher at infection sites of these bacteria (i.e. urine and skin) than in serum [22,23]. Thus, determination of the *in vivo* AUC/MPC ratio at each infection site is required to evaluate the practical likelihood of the emergence of fluoroquinolone-resistant mutants.

## Conclusions

In conclusion, the results of this study showed that the MPCs and MPC/MIC ratios of orbifloxacin against *E. coli*, *P. aeruginosa*, and *S. pseudintermedius* are mainly determined by the primary resistance mechanism of each bacterial species. Notably, *E. coli* and *P. aeruginosa*, which are representative Gram-negative bacteria frequently encountered in companion animal medicine, yielded markedly different MPCs of orbifloxacin. MPCs were also affected by the susceptibility (high or low) of the original isolate, especially in *S. pseudintermedius*. Therefore, the type of bacterial species and the fluoroquinolone susceptibility of the pathogen should be taken into consideration when using fluoroquinolone drugs such as orbifloxacin in canines.

## Competing interests

The authors declare no potential conflicts of interest with respect to the research, authorship, and publication of this article.

## Authors’ contributions

KH designed and TS carried out all the experiments. TS and KH contributed equally to this study. TS, KH, and YK were involved in preparation of the manuscript. KH drafted the manuscript. All authors read and approved the final version of the manuscript.
